# Detection of Unstable Carotid Plaque by Tissue Doppler Imaging and Contrast-Enhanced Ultrasound in a Patient with Recurrent Amaurosis Fugax

**DOI:** 10.1155/2013/354382

**Published:** 2013-01-09

**Authors:** Hagen Kunte, Ralph-Ingo Rückert, Charlotte Schmidt, Lutz Harms, Antje-Susanne Kasper, Rainer Hellweg, Maria Grigoryev, Thomas Fischer, Golo Kronenberg

**Affiliations:** ^1^Charité-Universitätsmedizin Berlin, Charitéplatz 1, 10117 Berlin, Germany; ^2^Franziskus-Krankenhaus, Budapester Straße 15-19, 10787 Berlin, Germany

## Abstract

Ultrasound (US) is one of the most important diagnostic tools available for the detection and evaluation of carotid stenosis. The case of a 70-year-old woman with recurrent right-sided amaurosis fugax presented here highlights the way in which tissue Doppler imaging (TDI) and contrast-enhanced US (CEUS) may aid in the diagnosis of carotid plaque vulnerability. Furthermore, the novel inverse fly-through technique was used for the three-dimensional visualization of the carotid stenosis.

## 1. Introduction

Stroke risk in patients with carotid stenosis may vary widely. The risk of ipsilateral ischemic stroke is increased in patients with a previous stroke. Furthermore, stroke risk increases progressively with the severity of carotid stenosis. The risk of a recurrent stroke or transient ischemic attack (TIA) is the greatest immediately after the initial ischemic event and decreases over time, as does the potential benefit which may be derived from carotid endarterectomy (CEA) [1]. The case presented here highlights the capabilities of modern vascular ultrasound techniques in the diagnosis and characterization of carotid artery stenosis.

## 2. Case Presentation

**Figure 1:**
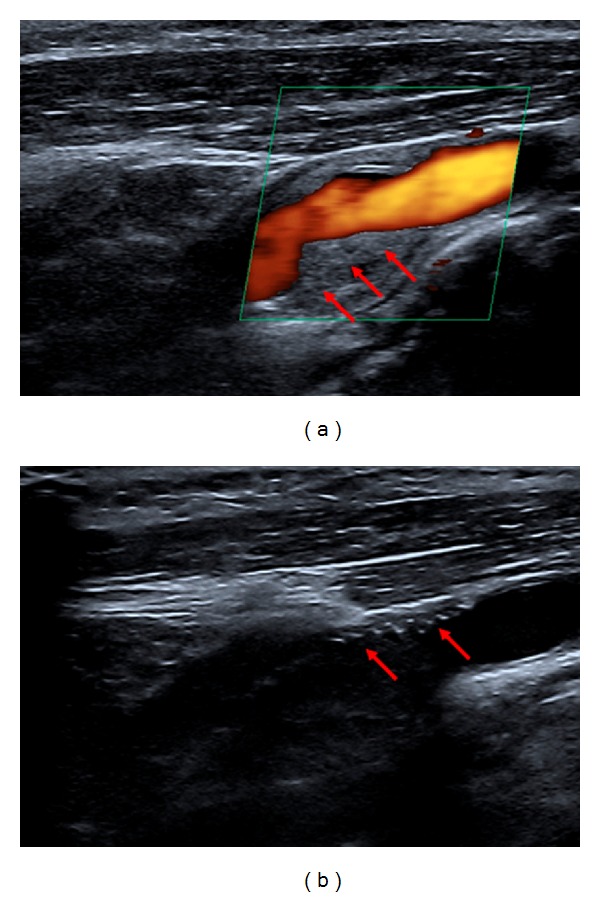
(a) B-mode and color Doppler ultrasound of right-sided internal carotid artery (ICA) stenosis. Red arrows indicate atherosclerotic ICA plaque. (b) Red arrows indicate the prosthetic patch used in CEA procedure eight years ago.

A 70-year-old woman was admitted after transient right monocular visual loss on the day of presentation and on the previous day. The medical history revealed that, eight years before, the patient had undergone right CEA for symptomatic stenosis of the internal carotid artery (ICA). Preceding that operation, the patient had suffered two TIAs with reversible paresis of the left arm. US examination on admission revealed a 60 percent right-sided ICA stenosis (Figure 1(a)). The prosthetic patch used to close the longitudinal arteriotomy could be clearly visualized (Figure 1(b)). Cranial MRI showed an old small subcortical infarct located in the right precentral gyrus as well as a very small area of restricted diffusion in the territory of the right middle cerebral artery (MCA). Cardiac workup, including electrocardiography, 24-hour Holter monitoring, and transthoracic echocardiography, did not reveal any abnormalities suggesting cardioembolic etiology.

**Figure 2:**
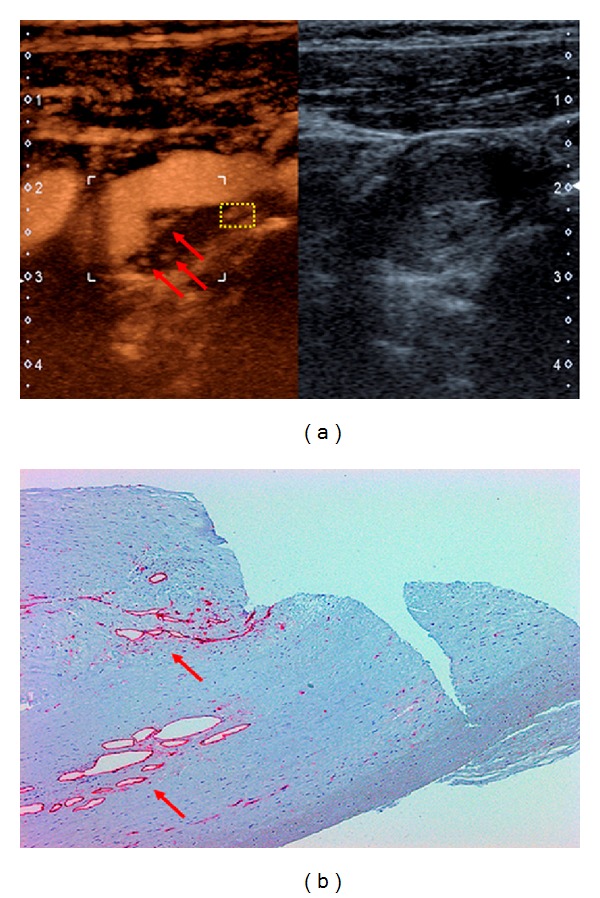
(a) Contrast-enhanced ultrasound (CEUS) of the carotid segment shown in Figure 1(a) (left). Intraplaque neovascularization is marked by red arrows. B-mode ultrasound of the same region (right). (b) Immunohistochemistry of CEA specimen corresponding to yellow-boxed area in (a). CD3 staining (red arrows) indicates plaque neovascularization.

**Figure 3:**
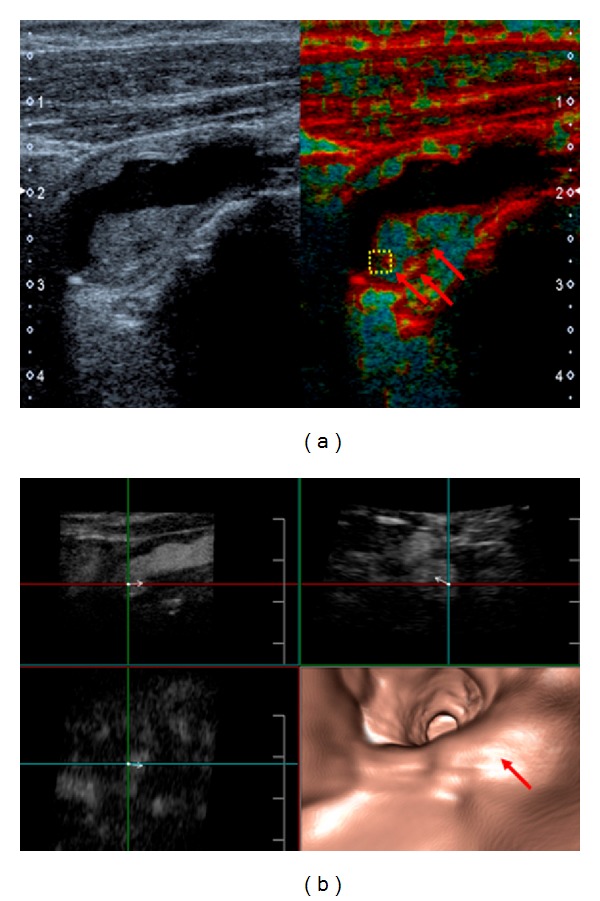
(a) B-mode and tissue Doppler imaging (TDI) of the patient's ICA stenosis. Brighter red coloring (arrows) indicates increased elastic deformability. (b) Inverse fly-through (FlyThru) technique with an intravessel view from ICA to the common carotid artery (bottom right). The red arrow marks the moderate ICA stenosis. The other three images show contrast-enhanced B-mode US used for the three-dimensional reconstruction of the inverse fly through. By changing the vectors, it is possible to visualize the stenotic segment from all imaginable directions.

An extended US examination including CEUS (Aplio 500, Toshiba, Otawa, Japan, in combination with the echo enhancer SonoVue, Bracco, Constance, Germany) demonstrated neovascularization of the carotid plaque (arrows in Figure 2(a)). Tissue Doppler imaging (TDI) revealed increased elastic deformability of the ICA in the vicinity of the area of plaque neovascularization indicating enhanced plaque elasticity (Figure 3(a)). The novel inverse fly-through US technique was used for three-dimensional imaging of the carotid stenosis and planning of potential surgery (Figure 3(b)).

**Figure 4:**
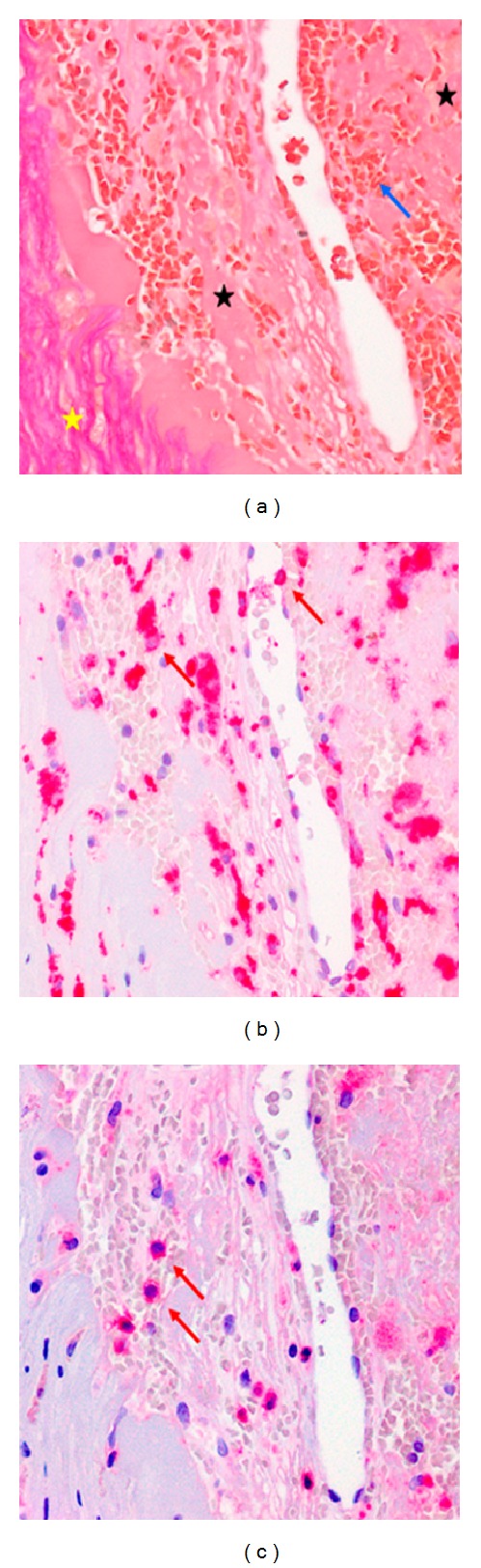
(a) Elastica van Gieson staining corresponding to yellow-boxed area in Figure 3(a). The blue arrow indicates area of acute intraplaque hemorrhage close to area of neoangiogenesis. Note that individual erythrocytes are clearly discernible. Black stars indicate thrombotic material. The yellow star indicates surrounding solid fibrotic tissue. (b) Immunohistochemistry of the same region as shown in Figure 3(a) with dense infiltrate of CD68-positive macrophages indicated by red arrows. (c) Immunohistochemistry of same region as shown in Figure 3(a) with infiltrate of CD3-positive T cells indicated by red arrows.

In summary, the most likely explanation of the recurrent transient monocular visual loss of our patient was amaurosis fugax secondary to right-sided 60 percent ICA stenosis. After weighing all risks and benefits, a recommendation for CEA was made. The procedure was performed promptly and went without complication. A good correlation was found between US findings and the microscopic examination of the CEA specimen (Figures 2(b), 4(a)–4(c)). In particular, histological analysis of the culprit lesion showed plaque neovascularization in the area previously identified by US (Figures 2(a) and 2(b)). In line with the US finding of increased plaque deformability (Figure 3(a)), histological examination revealed plaque neovascularization and invasion of inflammatory cells with acute intraplaque hemorrhage and intraplaque thrombus formation (Figures 4(a)–4(c)), most likely due to previous intraplaque bleeding. Meanwhile, the patient has been symptom free for over three months. She takes 100 mg q. d. acetylsalicylic acid for secondary prevention of stroke. Regular US follow-up examinations of the carotid and vertebral arteries have been scheduled.

## 3. Discussion

The recent advances in noninvasive carotid imaging promise the identification of new carotid plaque vulnerability markers. It is to be hoped that this will further improve the assessment of stroke risk associated with carotid stenosis.

Symptomatic carotid artery disease is associated with plaque neovascularization, intraplaque hemorrhage, and invasion of inflammatory cells. CEUS increasingly provides the means to quantify the degree of neovascularization of an atherosclerotic plaque. Since neovascularization is associated with an inflammatory infiltrate, CEUS may also indicate increased invasion of inflammatory cells [2–4].

Hypoechogenicity is already well established as a marker of high-risk carotid lesions [5]. TDI offers the additional opportunity to evaluate plaque characteristics such as tissue elasticity and the velocity at which tissue deformation occurs. The elastic properties of the tissue are shown as color-coded two-dimensional TDI images. Although more experience and data are necessary, the combination of several high-end US techniques will likely also facilitate the visualization of the transitional zone between an intraplaque thrombus and the less elastic fibrous tissue surrounding it. Finally, the three-dimensional inverse fly-through US technique allows video reconstruction of the contrast-enhanced blood vessel [6]. Importantly, by changing the vectors, the stenotic segment can be visualized from any direction (Figure 3(b)).

While there is already good data showing the value of CEUS in the diagnosis of carotid plaque neovascularisation, more work will be required to delineate the specific value of novel US techniques such as TDI and the three-dimensional fly-through technique.
